# Feasibility of celiac axis delineation and treatment on combined magnetic resonance imaging and linear accelerator systems

**DOI:** 10.1016/j.phro.2025.100768

**Published:** 2025-04-19

**Authors:** Sara N. Lim, Yirong Liu, Anugayathri Jawahar, Bharat B. Mittal, Tarita O. Thomas

**Affiliations:** a Department of Radiation Oncology, Northwestern University Feinberg School of Medicine, 251 E Huron St, Chicago, IL 60611, USA; b Department of Diagnostic Radiology, Northwestern University Feinberg School of Medicine, 676 N St Clair St, Chicago, IL 60611, USA

**Keywords:** Celiac plexus irradiation, Low-Field MRI, MR-Linac, Palliative care, Pancreatic cancer

## Abstract

Trials have been performed on irradiating celiac plexus for pancreatic cancer pain management. Images from a combined magnetic resonance imaging and linear accelerator system (MR-linac) for ten patients were assessed for delineation of celiac ganglia, aiming for smaller target volumes and reducing treatment risks *versus* standard linac-based treatments. MRI-linacs showed superior soft tissue contrast, enabling increased dose to ganglia while irradiating smaller target volumes *versus* regular linacs (median: 0.8 cm^3^ vs. 32.2 cm^3^, p < 0.05 for ten pairs of plans). While further studies are needed, MR-linac treatments could improve targeting precision, minimize dose to organs-at-risk and enhance effectiveness in palliative care.

## Highlights

• Combined magnetic resonance imaging and linear accelerator systems can visualize celiac ganglia.
• Due to the ability to see them, treatment volumes were greatly decreased.
• Resulting dose to organs-at-risk was minimized, while dose to ganglia increased.

## 1 Introduction

Pancreatic cancer is one of the deadliest cancers, ranking in the top ten leading causes of cancer-related deaths [1]. Significant number of patients (∼80 %) experience abdominal pain, severely impacting quality of life and requiring high doses of opioids [[2], [3], [4], [5]]. These have many side effects like constipation, sedation and nausea. Other approaches, such as celiac plexus block and neurolysis, have been explored to alleviate cancer-related abdominal pain [[6], [7], [8]]. Additionally, irradiating peripheral nerves for pain management has been applied to multiple sites, including brachial plexus and trigeminal nerve.

A phase II clinical trial was conducted to investigate the potential of radiotherapy in alleviating cancer-associated mid-back and epigastric pain [[9], [10], [11]]. Preliminary results demonstrated promising outcomes, with 84 % of patients reporting decrease in celiac pain three weeks after treatment [12,13]. Upon final evaluation, 58 % experienced at least partial pain relief [12]. Computed tomography (CT) images have limitations in delineating the celiac plexus, resulting in the need for a large surrogate structure based on anterior and medial aspects of the aorta from T12-L2 vertebrae [11]. This approach was employed as substitute for directly visualizing the celiac plexus, potentially resulting in over or under-treatment.

With superior soft tissue contrast, magnetic resonance imaging (MRI) is increasingly used for radiation treatment [[14], [15], [16]]. Currently available combined MRI and linear accelerator (MR-linac) systems enable more precise delineation of soft tissues, thus better dose delivery, and potential for daily adaptation and real-time motion management. For treatments in abdo-pelvic region, bowel loops are thus exposed to significantly less dose with adaptive radiotherapy [17]. This approach instills confidence in reducing treatment margins and delivering high dose to targets while reducing dose to critical structures [18]. In addition, MRI allows for better detection of celiac ganglia compared to CT, with studies showing that MRI can identify these structures with 98 % sensitivity [19,20].

The aim of this study was to assess feasibility of low-field MRI for celiac axis visualization and compare dose-volume profiles between CT-based surrogate and MRI-based celiac plexus radiation.

## 2 Materials and methods

The study was approved by Northwestern’s Institutional Review Board under protocol number STU00219731. It included ten patients with pancreatic cancer who underwent radiation therapy using a 0.35 T MR-linac system between May 2022 and July 2023.

### 2.1 Celiac ganglia and surrogate delineation

**Fig. 1:**
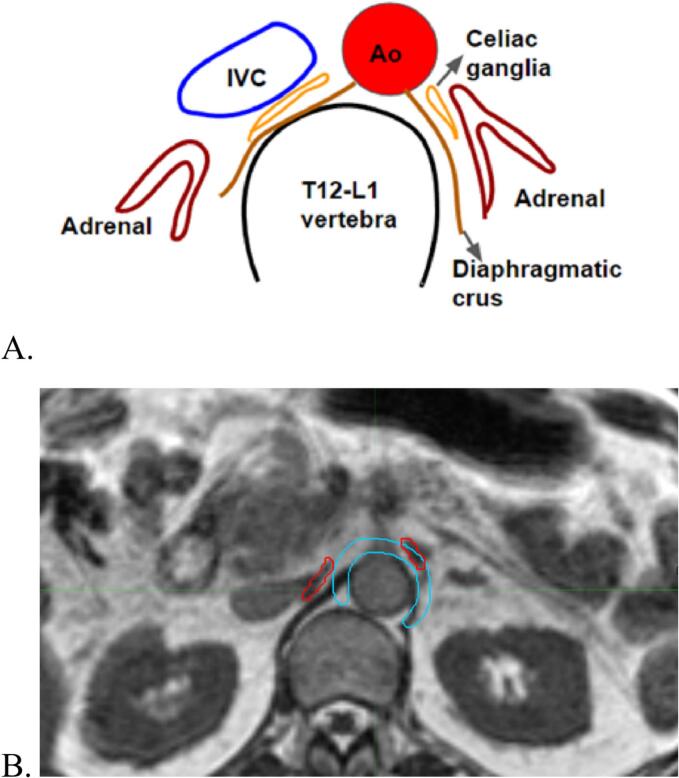
Celiac ganglia. **A.** Diagram of the location of the celiac ganglia (IVC = inferior vena cava; Ao = aorta); **B.** Axial MRI images showing the celiac plexus surrogate as contoured on MR for CT-based planning (blue contour around aorta) and bilateral ganglia as contoured for MRI plans (red contour, right: posterior to IVC; left: anterior to aorta).

Celiac ganglia are retroperitoneal structures located between T12 and L1 vertebral levels, with a diagram shown in Fig. 1A. They are identified as T1 hyperintense structures relative to liver and spleen [21]. On the right, these are seen at the level of the pancreas, between adrenal gland and crura of diaphragm, at a slightly superior angle due to left renal vein joining inferior vena cava, and partly or completely covered by inferior vena cava. On the left, it is seen close to abdominal aorta, between celiac and superior mesenteric arteries originating from aorta. Studies have shown different shapes of celiac ganglia: lamina, nodule- or sickle-shaped, with lamina-shape being most common [[21], [22], [23]]. Based on this knowledge, the ganglia were delineated by two radiation oncologists. The celiac ganglia were identified in all ten cases on images from 0.35 T MR-linac under guidance of a fellowship-trained abdominal MRI radiologist.

A surrogate for the celiac plexus was delineated according to the phase II celiac plexus radiation trial protocol [11]. In short, anterior and medial aspects of the aorta, from the levels of T12–L2 vertebrae inclusive were contoured as a surrogate structure. A representative case is presented in Fig. 1B, showing a comparison between celiac ganglia *versus* surrogate structure.

### 2.2 Treatment planning

Planning MRIs were obtained for each patient. Viewray™ treatment planning system (TPS) was used to generate ten pairs of plans: *MR plan* using ganglia contoured on MRI as target, and *CT plan* using surrogate structures contoured following the phase II trial. Organs-at-risk (OAR) volumes from patient treatments were used. Both plans were compared in terms of target coverage, volumes and OAR sparing following TG-101 report for single fraction treatments [24]. Since pain management is a consideration for these patients, total beam on time was considered during optimization.

MR plans were based on the precise contouring of celiac ganglia using MRI. A 5 mm expansion of celiac ganglia contour was created as planning target volume (PTV). A dose of 20 Gy was prescribed to PTV, with a simultaneous integrated boost of 25 Gy to celiac ganglia. A planning organ-at-risk volume consisting of 5 mm expansion of gastrointestinal structures was created and used to limit dose to OARs.

CT plans were based on the surrogate structure and followed the trial approach to minimize radiation exposure to gastrointestinal structures [11]. To summarize, PTV was a 5 mm expansion of the surrogate. Prescription was 25 Gy to surrogate and 20 Gy to PTV, with expansions and dose modifications to minimize exposure to surrounding organs. In cases of significant overlap with OARs, target coverage was sacrificed in favor of sparing OARs.

As proof of principle, a set of unilateral plans for one patient was created to target individual ganglion separately. For each unilateral plan, the contralateral ganglion was erased and PTV generated only on the ipsilateral ganglion. All other constraints followed the outline laid out above for MRI plans.

### 2.3 Statistical analysis

Mean doses and volumes for OARs and targets were obtained and recorded from TPS for all plans. Analysis was performed to compare the ten plans for each case using Wilcoxon matched-pairs signed rank test on Prism 10 software.

## 3 Results

MRI based plans resulted in significantly higher doses to celiac ganglia compared to CT plans (median dose of 25.7 Gy *versus* 21.3 Gy, p < 0.05) and also irradiated significantly smaller volumes compared to the CT-based plans, with a median volume of 0.8 cm^3^ for MR plans *versus* 32.2 cm^3^, p < 0.05 for CT plans (range: MRI: 0.3–4.6 cm^3^; CT: 22.1–36.2 cm^3^) (Table 1). Doses to OARs were consistently and significantly (p < 0.05) lower in MRI based plans (Table 1). Individual plan doses and volumes are presented in Table S1 and S2 of the supplement.

**Table 1:** **Mean dose** comparison for targets and organs-at-risk for CT vs MRI plans.

	**Mean dose to organ (Gy)**			
**Organ**	**Plan type**	**Range**	**Median**	***P***
**Celiac ganglia (target)**	CT	17.0–23.6	21.3	<0.05
	MRI	23.2–27.0	25.7	
**Right Kidney**	CT	1.1–2.8	2.3	<0.05
	MRI	0.3–1.0	0.5	
**Left Kidney**	CT	1.7–6.8	4.0	<0.05
	MRI	0.3–2.1	1.0	
**Spinal Cord**	CT	2.8–6.2	4.7	<0.05
	MRI	0.6–2.6	1.3	
**Stomach**	CT	3.8–6.7	4.8	<0.05
	MRI	0.7–2.8	1.2	
**Duodenum**	CT	2.7–7.1	4.9	<0.05
	MRI	0.2–1.9	0.7	
**Bowel**	CT	1.8–5.5	3.3	<0.05
	MRI	0.2–1.7	1.0	

MR-linacs also demonstrated the ability to target celiac ganglion on a unilateral basis. For patients with pain localized to one side, plans were created to deliver radiation only to the ipsilateral celiac ganglion. Selected dose results are presented in the supplement (Table S3) for representative unilateral plans, with a bilateral plan also presented for comparison. In all cases, TPS-reported beam-on times were under 25 mins.

## 4 Discussion

Results of this study supported use of MRI based radiation therapy for pain management in pancreatic cancer patients. MR-linacs could deliver higher doses to celiac ganglia while sparing surrounding organs. Increased precision could not only enhance therapeutic efficacy but also minimize dose to OARs.

Planning methods used in this study were largely based on stereotactic pancreatic cancer treatments on MR-linacs, wherein multiple studies [17,25,26] have shown the possibility to deliver extremely high, stereotactic doses to abdominal regions without causing severe side effects due to a combination of increased ability to visualize targets, motion management and daily adaptation [[27], [28], [29]]. The ability to use MRI for precise contouring also eliminated the need for surrogate structures as used in the phase II study [12], which can lead to over- or under-treatment. Another concern with using radiation to target the celiac plexus is its inability to differentiate the ipsilateral plexus, which could lead to unnecessary treatment of the unaffected side and increase risk of bilateral celiac plexus neuropathy. This can be particularly important in cases where pain is localized to one side of the body. This method could reduce risk of bilateral celiac plexus neuropathy, which can result in adverse effects like persistent diarrhea and postural hypotension. Besides, lower OARs dose might spare room for re-irradiation should the patient respond poorly to initial radiation or progress locally with symptoms. In the phase II trial with 18 patients, side effects included grade 2 diarrhea, vomiting, and nausea. Data on radiation-induced celiac plexus neuropathy is limited. However, complications from celiac plexus block and neurolysis procedures are common, with diarrhea occurring in 44–60 % and postural hypotension in 10–52 % of patients [30,31]. These issues are believed to result from unopposed parasympathetic activity [32,33]. Using MR images to create a radiation plan targeting only the ipsilateral celiac ganglion in unilateral cases may help preserve the contralateral plexus' function, reducing risk of bilateral blocking complications and minimizing radiation to OARs, particularly on contralateral sides.

Volumes used here for MR-plans were in the range of 0.3–4.6 cm^3^, which were comparable to targets typically treated with stereotactic radiosurgery (SRS)-capable linacs. However, performing an MR simulation would be necessary to obtain volumes used in this study, leading to delay between imaging and treatment, and potentially significant shifts in OAR positions. Adaptive treatment would then be necessary. In addition, cone beam CTs have very poor image quality thus requiring larger treatment regions. Even with more recent linacs that are capable of adaptive therapy, CT artifacts due to bowel gas and poor contrast make it very difficult to delineate OARs and ganglia. MR-linacs have limitations in both isocenter and beam placement locations and deliver plans with less ideal dose than SRS-capable linacs. The greatest justification for their use is in greatly decreased target volumes resulting in significantly lower OAR doses due to superior soft-tissue contrast, possibility of online adaptation and motion management [[27], [28], [29]]. In addition, TPS-reported beam-on times were comparable to other single-fraction, high dose plans on MR-linacs, translating to approximately 40–50 min total treatment time including imaging, plan adaptation and gating. This time frame is feasible for palliative treatment.

The study demonstrated promising results, particularly highlighting the limitations of using surrogate structures as in traditional CT-based radiation plans. This led to larger treatment volumes, which increased radiation exposure to healthy tissues. Moreover, dose delivered to celiac ganglia was often suboptimal, as evidenced by the lower doses delivered in the CT plans. Due to higher doses to celiac ganglia in MR plans compared to CT plans, and lower doses to OARs, we hypothesize that MR plans could achieve similar pain control with potentially fewer side effects. While MR plans target only the ganglia, pain pathways from tumors with perivascular invasion lead to them, therefore, targeting them alone may help control pain [34]. This study has a few limitations: Sample size was small and was a planning study without patient outcomes; The hypothesis requires testing through a prospective clinical trial; Since all patients had pancreatic cancer, findings may not be easily generalized to other cancer types, though volumetric and dose profiles for celiac plexus treatment should be consistent across different cancers.

In conclusion, MR plans for celiac plexus radiation based on low-field MR-linac images enhanced the therapeutic ratio by delivering targeted dose to celiac plexus and lower dose to surrounding OARs. Radiation to ipsilateral celiac plexus was shown to be feasible in MR systems, offering potential benefits in reducing complications. Prospective evaluation of MR based celiac plexus radiation to validate these potential benefits is still needed.

## Declaration of competing interest

The authors declare that they have no known competing financial interests or personal relationships that could have appeared to influence the work reported in this paper.

## References

[1] Siegel R.L., Miller K.D., Wagle N.S., Jemal A. (2023). Cancer statistics, 2023. CA Cancer J Clin.

[2] Idachaba S., Dada O., Abimbola O., Olayinka O., Uma A., Olunu E. (2019). A Review of Pancreatic Cancer: Epidemiology, Genetics, Screening, and Management. Open Access Maced. J Med Sci.

[3] Koulouris A.I., Banim P., Hart A.R. (2017). Pain in Patients with Pancreatic Cancer: Prevalence, Mechanisms, Management and Future Developments. Dig Dis Sci.

[4] Zylberberg H.M., Woodrell C., Rustgi S.D., Aronson A., Kessel E., Amin S. (2022). Opioid Prescription Is Associated With Increased Survival in Older Adult Patients With Pancreatic Cancer in the United States: A Propensity Score Analysis. JCO Oncol Pract.

[5] Scarborough B.M., Smith C.B. (2018). Optimal pain management for patients with cancer in the modern era. CA Cancer J Clin.

[6] Okita M., Otani K., Gibo N., Matsui S. (2022). Systematic review and meta-analysis of celiac plexus neurolysis for abdominal pain associated with unresectable pancreatic cancer. Pain Pract.

[7] Wong G.Y., Schroeder D.R., Carns P.E., Wilson J.L., Martin D.P., Kinney M.O. (2004). Effect of neurolytic celiac plexus block on pain relief, quality of life, and survival in patients with unresectable pancreatic cancer: a randomized controlled trial. JAMA.

[8] Arcidiacono P.G., Calori G., Carrara S., McNicol E.D., Testoni P.A. (2011). Celiac plexus block for pancreatic cancer pain in adults. Cochrane Database Syst Rev.

[9] Tello Valverde C.P., Ebrahimi G., Sprangers M.A., Pateras K., Bruynzeel A.M.E., Jacobs M. (2024). Impact of Short-Course Palliative Radiation Therapy on Pancreatic Cancer-Related Pain: Prospective Phase 2 Nonrandomized PAINPANC Trial. Int J Radiat Oncol Biol Phys.

[10] Li C.P., Chao Y., Chi K.H., Chan W.K., Teng H.C., Lee R.C. (2003). Concurrent chemoradiotherapy treatment of locally advanced pancreatic cancer: gemcitabine versus 5-fluorouracil, a randomized controlled study. Int J Radiat Oncol Biol Phys.

[11] Jacobson G., Fluss R., Dany-BenShushan A., Golan T., Meron T., Zimmermann C. (2022). Coeliac plexus radiosurgery for pain management in patients with advanced cancer: study protocol for a phase II clinical trial. BMJ Open.

[12] Lawrence Y.R., Miszczyk M., Dawson L.A., Diaz Pardo D.A., Aguiar A., Limon D. (2024). Celiac plexus radiosurgery for pain management in advanced cancer: a multicentre, single-arm, phase 2 trial. Lancet Oncol.

[13] Hammer L., Hausner D., Ben-Ayun M., Shacham-Shmueli E., Morag O., Margalit O. (2022). Single-Fraction Celiac Plexus Radiosurgery: A Preliminary Proof-of-Concept Phase 2 Clinical Trial. Int J Radiat Oncol Biol Phys.

[14] Chandarana H., Wang H., Tijssen R.H.N., Das I.J. (2018 Dec). Emerging role of MRI in radiation therapy. J Magn Reson Imaging.

[15] Das I.J., McGee K.P., Tyagi N., Wang H. (2019). Role and future of MRI in radiation oncology. Br J Radiol.

[16] Pollard J.M., Wen Z., Sadagopan R., Wang J., Ibbott G.S. (2017). The future of image-guided radiotherapy will be MR guided. Br J Radiol.

[17] Cuccia F., Rigo M., Gurrera D., Nicosia L., Mazzola R., Figlia V. (2021). Mitigation on bowel loops daily variations by 1.5-T MR-guided daily-adaptive SBRT for abdomino-pelvic lymph-nodal oligometastases. J Cancer Res Clin Oncol.

[18] Lagendijk J.J., Raaymakers B.W., Raaijmakers A.J., Overweg J., Brown K.J., Kerkhof E.M. (2008 Jan). MRI/linac integration. Radiother Oncol.

[19] Krohn T, Verburg FA, Pufe T, Neuhuber W, Vogg A, Heinzel A, et al. [(68)Ga]PSMA-HBED uptake mimicking lymph node metastasis in coeliac ganglia: an important pitfall in clinical practice. Eur J Nucl Med Mol Imaging. 2015;42:210-4. https://doi.org/ 10.1007/s00259-014-2915-3. 10.1007/s00259-014-2915-3 25248644

[20] Liu S., Fu W., Liu Z., Liu M., Ren R., Zhai H. (2016 Oct). MRI-guided celiac plexus neurolysis for pancreatic cancer pain: Efficacy and safety. J Magn Reson Imaging.

[21] Zhang X.M., Zhao Q.H., Zeng N.L., Cai C.P., Xie X.G., Li C.J. (2006). The celiac ganglia: anatomic study using MRI in cadavers. AJR Am J Roentgenol.

[22] Wang Z.J., Webb E.M., Westphalen A.C., Coakley F.V., Yeh B.M. (2010). Multi-detector row computed tomographic appearance of celiac ganglia. J Comput Assist Tomogr.

[23] Bialek E.J., Malkowski B. (2019). Celiac ganglia: can they be misinterpreted on multimodal 68Ga-PSMA-11 PET/MR?. Nucl Med Commun.

[24] Benedict S.H., Yenice K.M., Followill D., Galvin J.M., Hinson W., Kavanagh B. (2010). Stereotactic body radiation therapy: The report of AAPM Task Group 101. Med Phys,.

[25] Grimbergen G., Eijkelenkamp H., Snoeren L.M.W., Bahij R., Uffe B., van der Bijl E. (2024). Treatment planning for MR-guided SBRT of pancreatic tumors on a 1.5 T MR-Linac: A global consensus protocol. Clin Transl. Radiat Oncol.

[26] Grimbergen G., Eijkelenkamp H., van Vulpen J.K., van de Ven S., Raaymakers B.W., Intven M.P.W. (2023). Feasibility of online radial magnetic resonance imaging for adaptive radiotherapy of pancreatic tumors. Phys Imaging Radiat Oncol.

[27] Jassar H., Tai A., Chen X., Keiper T.D., Paulson E., Lathuilere F. (2023). Real-time motion monitoring using orthogonal cine MRI during MR-guided adaptive radiation therapy for abdominal tumors on 1.5T MR-Linac. Med Phys.

[28] Fast M.F., Cao M., Parikh S.-J. (2024). Intrafraction Motion Management With MR-Guided Radiation Therapy. Semin Radiat Oncol.

[29] Daly M., McDaid L., Anandadas C., Brocklehurst A., Choudhury A., McWilliam A. (2024). Impact of motion management strategies on abdominal organ at risk delineation for magnetic resonance-guided radiotherapy. Phys Imaging Radiat Oncol.

[30] Eisenberg E., Carr D.B., Chalmers T.C. (1995). Neurolytic celiac plexus block for treatment of cancer pain: a meta-analysis. Anesth Analg.

[31] Mohamed R.E., Amin M.A., Omar H.M. (2017). Computed tomography-guided celiac plexus neurolysis for intractable pain of unresectable pancreatic cancer. Egypt J Radiol Nucl Med.

[32] Kambadakone A., Thabet A., Gervais D.A., Mueller P.R., Arellano R.S. (2011). CT-guided celiac plexus neurolysis: a review of anatomy, indications, technique, and tips for successful treatment. Radiographics.

[33] Yang A., Brown J., Mak E. (2016). Persistent Diarrhea after Celiac Plexus Block in a Pancreatic Cancer Patient: Case Report and Literature Review. J Palliat Med.

[34] Cornman-Homonoff J., Holzwanger D.J., Lee K.S., Madoff D.C., Li D. (2017). Celiac Plexus Block and Neurolysis in the Management of Chronic Upper Abdominal Pain. Semin Intervent Radiol.

