# Supplementary material for: Feasibility of celiac axis delineation and treatment on combined magnetic resonance imaging and linear accelerator systems
Source: Phys Imaging Radiat Oncol. 2025 Apr 19;34:100768. doi: 10.1016/j.phro.2025.100768 (PMC12051637; doi:10.1016/j.phro.2025.100768)
Supplement: Supplementary Data 1 [file mmc1.docx]

Table 1: Treatment dose and volume comparison between CT and MRI based celiac radiation plans

|  | **Mean Dose (Gy)** | | **Volume (cm^3^)** | |
| --- | --- | --- | --- | --- |
| **Pt ID** | Celiac ganglia  CT plans | Celiac ganglia  MRI plans | Celiac surrogate  CT plans | Celiac ganglia  MRI plans |
| **1** | 19.5 | 24.6 | 34.8 | 0.6 |
| **2** | 20.5 | 24.3 | 32.5 | 1.2 |
| **3** | 22.0 | 25.2 | 31.9 | 0.5 |
| **4** | 23.6 | 24.6 | 39.4 | 1.0 |
| **5** | 17.0 | 27.0 | 22.1 | 0.3 |
| **6** | 22.9 | 26.2 | 31.8 | 0.6 |
| **7** | 23.6 | 26.3 | 36.2 | 1.2 |
| **8** | 22.3 | 26.4 | 36.1 | 1.1 |
| **9** | 17.9 | 23.2 | 27.0 | 0.5 |
| **10** | 18.9 | 26.4 | 28.6 | 4.6 |
| **Median** | 21.3 | 25.7 | 32.2 | 0.8 |
| ***P*** |  | <0.05 |  | <0.05 |

Table 2: Mean dose to organs-at-risks for both CT and MR plans. N/a refers to organs that have been excised from patients

| **Mean organ dose (Gy)** | | | | | | | | | | | | |
| --- | --- | --- | --- | --- | --- | --- | --- | --- | --- | --- | --- | --- |
| **Pt ID** | **Right Kidney** | | **Left Kidney** | | **Spinal Cord** | | **Stomach** | | **Duodenum** | | **Bowel** | |
|  | **CT** | **MRI** | **CT** | **MRI** | **CT** | **MRI** | **CT** | **MRI** | **CT** | **MRI** | **CT** | **MRI** |
| **1** | 2.6 | 0.2 | 3.7 | 0.4 | 4.6 | 0.6 | 4.8 | 0.7 | 2.7 | 0.2 | 2.0 | 0.3 |
| **2** | 1.1 | 0.2 | 2.8 | 0.3 | 4.9 | 1.3 | 4.0 | 2.0 | 2.6 | 0.2 | 1.8 | 0.2 |
| **3** | 2.8 | 1.0 | 6.8 | 1.6 | 5.9 | 1.4 | 5.6 | 1.1 | 4.9 | 1.4 | 5.5 | 1.7 |
| **4** | 2.3 | 0.6 | 5.5 | 1.7 | 2.8 | 0.9 | 4.4 | 0.6 | n/a | n/a | 3.9 | 1.4 |
| **5** | n/a | n/a | 4.2 | 0.3 | 4.7 | 1.0 | 6.7 | 1.6 | 7.1 | 0.3 | 2.8 | 0.2 |
| **6** | 2.5 | 0.5 | 5.8 | 0.9 | 4.3 | 1.2 | 5.5 | 1.3 | 4.5 | 0.3 | 1.6 | 0.3 |
| **7** | n/a | n/a | 3.7 | 1.1 | 9.8 | 0.9 | 4.8 | 1.0 | 5.8 | 1.5 | 3.9 | 0.9 |
| **8** | 2.1 | 0.7 | 3.5 | 2.1 | 6.2 | 2.6 | 3.8 | 1.1 | 6.4 | 2.1 | 3.7 | 1.1 |
| **9** | 1.9 | 0.3 | 4.5 | 1.1 | 4.2 | 1.6 | 5.2 | 1.9 | 6.7 | 0.7 | 4.7 | 1.1 |
| **10** | n/a | n/a | 1.7 | 0.5 | 3.6 | 1.6 | 4.0 | 2.8 | 4.3 | 1.9 | 2.6 | 1.0 |
| **Median** | 2.3 | 0.5 | 4.0 | 1.0 | 4.7 | 1.3 | 4.8 | 1.2 | 4.9 | 0.7 | 3.3 | 1 |
| ***P*** |  | <0.05 |  | <0.05 |  | <0.05 |  | <0.05 |  | <0.05 |  | <0.05 |

Table 3: Mean, max doses and selected TG-101 results for sample right vs left vs bilateral plans. The TG-101 single fraction dose constraints are listed in the last column.

|  | **Right Ganglion** | | | **Left Ganglion** | | | **Bilateral ganglia** | | | **Selected TG-101 constraints** |
| --- | --- | --- | --- | --- | --- | --- | --- | --- | --- | --- |
|  | **Mean (Gy)** | **Max (Gy)** | **TG-101** | **Mean (Gy)** | **Max (Gy)** | **TG-101** | **Mean (Gy)** | **Max (Gy)** | **TG-101** |  |
| **Rt Kidney** | 0.9 |  | 0 cm^3^ | 0.4 |  | 0 cm^3^ | 1 |  | 0 cm^3^ | 200 cm^3^ < 8.4 Gy |
| **Lt Kidney** | 0.7 |  | 0 cm^3^ | 1.3 |  | 0 cm^3^ | 1.9 |  | 0 cm^3^ | 200 cm^3^ < 8.4 Gy |
| **Spinal Canal** | 1.1 | 5.2 | 0 cm^3^ | 0.8 | 4.7 | 0 cm^3^ | 1.7 | 8.1 | 0 cm^3^ | 0.35 cm^3^ at 10 Gy |
| **Stomach** | 0.6 | 5.5 | 0 cm^3^ | 1.1 | 9.5 | 0 cm^3^ | 1.3 | 8.5 | 0 cm^3^ | 0.03 cm^3^ at 12.4 Gy |
| **Duodenum** | 1.3 | 8.2 | 0 cm^3^ | 0.6 | 4.2 | 0 cm^3^ | 1.6 | 7.9 | 0 cm^3^ | 0.03 cm^3^ at 12.4 Gy |
| **Bowel** | 0.5 | 9.2 | 0 cm^3^ | 1 | 7.8 | 0 cm^3^ | 1.2 | 10.1 | 0 cm^3^ | 5 cm^3^ @ 11.9 Gy |
| **Liver** | 1 |  | 10.6 cm^3^ | 0.5 |  | 0 cm^3^ | 1.3 |  | 15.1 cm^3^ | <700 cm^3^ at 10 Gy |
| **Contralateral ganglion*** | 5.1 |  | 6.1 | 3.5 |  | 3.9 | n/a, below refers to bilateral ganglia | | | Max < 17.5 Gy* |
| **Ipsilateral ganglion** | 25.6 |  | 95.3 | 25.6 |  | 98.1 | 25.5 |  | 95.7 | % at 25 Gy |
| * Contralateral celiac ganglia have no specific TG-101 constraints. Constraint listed here is based on brachial plexus dose limits | | | | | | | | | | |
