# Supplementary material for: Feasibility of celiac axis delineation and treatment on combined magnetic resonance imaging and linear accelerator systems
Source: Phys Imaging Radiat Oncol. 2025 Apr 19;34:100768. doi: 10.1016/j.phro.2025.100768 (PMC12051637; doi:10.1016/j.phro.2025.100768)
Supplement: Supplementary Data 2 [file mmc2.pdf]

Table 1: Treatment dose and volume comparison between CT and MRI based celiac radiation plans

	**Mean Dose (Gy)**		**Volume (cm^3)**	
	Celiac ganglia	Celiac ganglia	Celiac surrogate	Celiac ganglia
**Pt ID**	CT plans	MRI plans	CT plans	MRI plans
**1**	19.5	24.6	34.8	0.6
**2**	20.5	24.3	32.5	1.2
**3**	22.0	25.2	31.9	0.5
**4**	23.6	24.6	39.4	1.0
**5**	17.0	27.0	22.1	0.3
**6**	22.9	26.2	31.8	0.6
**7**	23.6	26.3	36.2	1.2
**8**	22.3	26.4	36.1	1.1
**9**	17.9	23.2	27.0	0.5
**10**	18.9	26.4	28.6	4.6
**Median**	21.3	25.7	32.2	0.8
P		<0.05		<0.05

Table 2: Mean dose to organs-at-risks for both CT and MR plans. N/a refers to organs that have been excised from patients

	Mean organ dose (Gy)											
	**Right Kidney**		**Left Kidney**		**Spinal Cord**		**Stomach**		**Duodenum**		**Bowel**	
**Pt ID**	**CT**	**MRI**	**CT**	**MRI**	**CT**	**MRI**	**CT**	**MRI**	**CT**	**MRI**	**CT**	**MRI**
**1**	2.6	0.2	3.7	0.4	4.6	0.6	4.8	0.7	2.7	0.2	2.0	0.3
**2**	1.1	0.2	2.8	0.3	4.9	1.3	4.0	2.0	2.6	0.2	1.8	0.2
**3**	2.8	1.0	6.8	1.6	5.9	1.4	5.6	1.1	4.9	1.4	5.5	1.7
**4**	2.3	0.6	5.5	1.7	2.8	0.9	4.4	0.6	n/a	n/a	3.9	1.4
**5**	n/a	n/a	4.2	0.3	4.7	1.0	6.7	1.6	7.1	0.3	2.8	0.2
**6**	2.5	0.5	5.8	0.9	4.3	1.2	5.5	1.3	4.5	0.3	1.6	0.3
**7**	n/a	n/a	3.7	1.1	9.8	0.9	4.8	1.0	5.8	1.5	3.9	0.9
**8**	2.1	0.7	3.5	2.1	6.2	2.6	3.8	1.1	6.4	2.1	3.7	1.1
**9**	1.9	0.3	4.5	1.1	4.2	1.6	5.2	1.9	6.7	0.7	4.7	1.1
**10**	n/a	n/a	1.7	0.5	3.6	1.6	4.0	2.8	4.3	1.9	2.6	1.0
**Median**	2.3	0.5	4.0	1.0	4.7	1.3	4.8	1.2	4.9	0.7	3.3	1
P		<0.05		<0.05		<0.05		<0.05		<0.05		<0.05

Table 3: Mean, max doses and selected TG-101 results for sample right vs left vs bilateral plans.

The TG-101 single fraction dose constraints are listed in the last column.

	Right Ganglion			Left Ganglion			Bilateral ganglia			Selected TG-101 constraints
	**Mean (Gy)**	**Max (Gy)**	**TG-101**	**Mean (Gy)**	**Max (Gy)**	**TG-101**	**Mean (Gy)**	**Max (Gy)**	**TG-101**	
**Rt Kidney**	0.9		0 cm^3	0.4		0 cm^3	1		0 cm^3	200 cm^3 < 8.4 Gy
**Lt Kidney**	0.7		0 cm^3	1.3		0 cm^3	1.9		0 cm^3	200 cm^3 < 8.4 Gy
**Spinal Canal**	1.1	5.2	0 cm^3	0.8	4.7	0 cm^3	1.7	8.1	0 cm^3	0.35 cm^3 at 10 Gy
**Stomach**	0.6	5.5	0 cm^3	1.1	9.5	0 cm^3	1.3	8.5	0 cm^3	0.03 cm^3 at 12.4 Gy
**Duodenum**	1.3	8.2	0 cm^3	0.6	4.2	0 cm^3	1.6	7.9	0 cm^3	0.03 cm^3 at 12.4 Gy
**Bowel**	0.5	9.2	0 cm^3	1	7.8	0 cm^3	1.2	10.1	0 cm^3	5 cm^3 @ 11.9 Gy
**Liver**	1		10.6 cm^3	0.5		0 cm^3	1.3		15.1 cm^3	<700 cm^3 at 10 Gy
**Contralateral ganglion***	5.1		6.1	3.5		3.9	n/a, below refers to bilateral ganglia			Max < 17.5 Gy*
**Ipsilateral ganglion**	25.6		95.3	25.6		98.1	25.5		95.7	% at 25 Gy

* Contralateral celiac ganglia have no specific TG-101 constraints. Constraint listed here is based on brachial plexus dose limits
